# Stabilization of Sunflower Oil with Biologically Active Compounds from Berries

**DOI:** 10.3390/molecules28083596

**Published:** 2023-04-20

**Authors:** Aliona Ghendov-Mosanu, Violina Popovici, Cristina Gabriela Constantinescu (Pop), Olga Deseatnicova, Rodica Siminiuc, Iurie Subotin, Raisa Druta, Adela Pintea, Carmen Socaciu, Rodica Sturza

**Affiliations:** 1 Faculty of Food Technology, Technical University of Moldova, MD-2045 Chisinau, Moldova; aliona.mosanu@tpa.utm.md (A.G.-M.); violina.popovici@toap.utm.md (V.P.); olga.deseatnicova@toap.utm.md (O.D.); rodica.siminiuc@adm.utm.md (R.S.); iurie.subotin@fta.utm.md (I.S.); raisa.druta@fta.utm.md (R.D.); 2 Faculty of Food Engineering, Ştefan cel Mare University of Suceava, 720229 Suceava, Romania; gabriela.constantinescu@fia.usv.ro; 3 Faculty of Veterinary Medicine, University of Agricultural Sciences and Veterinary Medicine, 400374 Cluj-Napoca, Romania; apintea@usamvcluj.ro (A.P.); carmen.socaciu@usamvcluj.ro (C.S.)

**Keywords:** sunflower oil, carotenoids, sea buckthorn, rose hips, lipid oxidation, oxidative stability

## Abstract

Sunflower oil (*Helianthus annuus*) contains a rich concentration of polyunsaturated fatty acids, which are susceptible to rapid oxidative processes. The aim of this study was to evaluate the stabilizing effect of lipophilic extracts from two types of berries, sea buckthorn and rose hips, on sunflower oil. This research included the analysis of sunflower oil oxidation products and mechanisms, including the determination of chemical changes occurring in the lipid oxidation process via LC-MS/MS using electrospray ionization in negative and positive mode. Pentanal, hexanal, heptanal, octanal, and nonanal were identified as key compounds formed during oxidation. The individual profiles of the carotenoids from sea buckthorn berries were determined using RP-HPLC. The influence of the carotenoid extraction parameters ascertained from the berries on the oxidative stability of sunflower oil was analyzed. The dynamics of the accumulation of the primary and secondary products of lipid oxidation and the variation of the carotenoid pigment content in the lipophilic extracts of sea buckthorn and rose hips during storage demonstrated good stability at 4 °C in the absence of light for 12 months. The experimental results were applied to mathematical modeling using fuzzy sets and mutual information analysis, which allowed for the prediction of the oxidation of sunflower oil.

## 1. Introduction

A permanent concern in the modern food industry is to ensure an optimal shelf life of food products. The main factor that leads to food spoilage is oxidation [1,2]. Oxidation is a chemical process involving the modification of fatty acids, amino acids, and vitamins, which, in turn, affects the organoleptic and nutritional characteristics of food [3,4].

The oxidation of lipids in food is a complex process that is influenced by various factors, such as the chemical structure of the food; the food’s physical condition; the quantity and quality of substances that act as antioxidants in food; and the processing, packaging, and storage conditions of the food. Lipids are an easily alterable fraction of food, so their storage conditions largely depend on their nature and concentration. The consequences of this process, which are precipitated by the degradation of some easily oxidizable constituents of the lipid fraction in food, can include the appearance of a rancid odor, color changes, and, in some cases, changes in food texture, which negatively influence the sensory parameters of food [5]. The nutritional value of foods subjected to oxidative degradation of their lipid fraction can also be affected to a considerable extent. However, the most important risk is the ingestion of lipid oxidation products because they present enormous toxicological risks and, in the case of long-term use, can lead to the appearance of degenerative pathologies such as arteriosclerosis, cancer, etc.

After soybean oil, sunflower oil (*Helianthus annuus*) ranks second in the world in terms of the most-produced edible oils and is rightly considered to number among the preeminent vegetable oils for the human diet due to its nutritional value [6]. Sunflower oil contains a high concentration of polyunsaturated fatty acids (especially oleic and linoleic acids) that contribute to lowering cholesterol and reducing the risk of heart disease. However, polyunsaturated fatty acids are the site of rapid oxidative processes, especially in the presence of oxygen and light. Plant sterols or phytosterols are compounds with proven health benefits. Their richest natural sources are vegetable oils. In crude sunflower vegetable oil, phospholipids represent 0.8–1.2% of the total lipid fraction [7]. Unsaturated fatty acids esterified in phospholipids are major targets for oxidation. The hydrogen atoms on the methylene groups adjacent to the double bonds (allylic hydrogen atoms) present low C-H bond energies, and those located on the methylene between two double bonds (hydrogen bis- allylic) have even lower C-H bond energies. This fact allows them to be easily abstracted by reactive radical species, leading to the formation of phospholipid radical species with the radical centered on the allylic carbon atom. Refinement and deodorization processes considerably decrease the content of phospholipids (0.1–0.2%). This increases the stability of the oils upon oxidation but decreases their biological value [8].

In biological materials, lipids are protected from oxidation by the presence of antioxidants and cell membranes, which reduce the access of oxidants to easily oxidizable fractions [3]. In food, reducing the impact of lipid oxidation can only be ensured by the use of appropriate packaging and by the presence of antioxidants, which block the propagation or decomposition of hydroperoxides. Food products usually contain antioxidants of a synthetic origin (propyl gallate—E-311 or octyl gallate—E-312; butylhydroxyanisole (BHA)—E-320; etc.), but their effect on human health is debatable. One of the current strategies used in the food industry with the aim of inhibiting the lipid oxidation process is the use of natural antioxidants, which are compounds that increase the shelf life of food products following the oxidation process [9]. Lipophilic extracts from vegetable powders of berries, which are rich in biologically active compounds, are of particular interest [10,11,12].

Tocopherols are the most important antioxidants present in edible oil. Sunflower oil contains a high concentration of tocopherols (648.9 ppm). The refinement process, especially deodorization, reduces tocopherol content [13,14,15]. Carotenoids are a group of tetraterpenoids that are formed from isoprenoid units and have conjugated double bonds. In the co-presence of chlorophylls, β-carotene reduces the oxidation of edible oil by inhibiting ^1^O_2_ [16,17]. Sea buckthorn (*Hippophae rhamnoides* L.) is a valuable plant due to its medicinal and nutritional potential, as it is a good source of bioactive compounds such as vitamins (C, E, and K; riboflavin; and folic acid), carotenoids (α-, β-, and δ-carotene; lycopene), tocopherols, phytosterols, organic acids (malic acid, oxalic acid, and citric acid), polyunsaturated fatty acids, and some essential amino acids [18,19,20]. Rose hip berries (*Rosa canina* L.) are rich in polyphenols, carotenoids, triterpenic acids, essential fatty acids, galactolipids, folates, vitamins C and E, minerals (Ca, Mg, K, S, Si, Se, Mn, and Fe), etc. [21]. It has been reported that rose hips can be used not only for direct consumption but also for the extraction of bioactive compounds (BC) [22].

The aim of this study was to assess the stabilizing effect of lipophilic extracts from berries-sea buckthorn and rose hips on sunflower oil. Firstly, we determined the optimal conditions for obtaining the lipophilic extracts from the berries. Secondly, an analysis of the products and mechanisms of sunflower oil oxidation was conducted. This included determining the chemical changes that occur in the process of a forced lipid oxidation analysis of the evolution of the stability parameters of the stabilized oils over a period of 12 months, mathematically modeling the oxidation processes, and determining the influencing factors.

## 2. Results and Discussion

### 2.1. Analysis of Oxidation Products and Kinetics of Forced Autoxidation of Sunflower Oil

The oxidation of food products cannot be completely avoided by limiting the access of oxygen, light, or other reactive oxygen species. The first stage of lipid oxidation consists of the formation of free radicals, which depends on the availability of oxygen, light, and temperature [23]. The first products of lipid oxidation are peroxides, which are primary oxidation products that are unstable and rapidly break down into secondary products. The most likely pathway for the decomposition of hydroperoxide is a homolytic cleavage of the bond between the oxygen atoms, whereby alkoxy and hydroxy radicals are produced. The alkoxy radical then undergoes homolytic β-scission of the carbon–carbon bond and produces oxo compounds and saturated or unsaturated alkyl radicals. After electron rearrangement, hydroxyl radical addition, or hydrogen transfer, the final lipid oxidation byproducts are mostly low-molecular-weight aldehydes, ketones, alcohols, short-chain hydrocarbons, etc. Most of the breakdown products of hydroperoxides induce a rancid flavor in the oxidized edible oil [24].

As a result of testing via liquid chromatography coupled with tandem mass spectrometry, several products of lipid oxidation were identified (Table 1). The results demonstrate the diversity of lipid oxidation products formed by oxygen addition (epoxidation and OH addition) and oxidative cleavage (aldehydes and corresponding carboxylic acids). The rate of lipid oxidation is determined by a complex set of factors that include the fatty acid composition of the analyzed lipids or the presence of antioxidant compounds. The identification of lipid peroxidation products (LPP) allowed us to study the lipid oxidation mechanisms and the quantitative evolution of the lipid oxidation products formed during the oxidation process.

The main high-molecular-weight lipid oxidation products identified using ESI ionization in positive mode are as follows: PC (1:0/20:4 <oxo@C1>) with an *m*/*z* = 829.6680; PC (P-18:1/5:0 <oxo@C5>) with an *m*/*z* = 863.5298; and PC (P-18:0/11:2 <OH@C5>) with an *m*/*z* = 959.5877. Derivatization was confirmed by the presence of characteristic peaks: *m*/*z* = 262.1074 and *m*/*z* = 244.0967. The high-molecular-weight lipid oxidation products identified were formed as a result of carbon chain breakage or the addition of a hydroxyl group.

High-molecular-weight lipid oxidation products were identified via ESI ionization in negative mode. Ionized compounds were formed via formate addition ([M + HCOO]^−^) or deprotonation ([M-H]). The main compounds identified are as follows: 1-LysoPC with an *m*/*z* = 588.3312; PC (P-18:0/20:4 <epoxy@sn2>) with an *m*/*z* = 854.5920; PC (P-18:0/20:4 <OH@C11>) with an *m*/*z* = 854.5887; and PC(P-18:0/7:1 <oxo@C7>) with an *m*/*z* = 676.4197. High-molecular-weight LPPs can be identified without a derivatization step. The listed compounds were abundantly formed via the truncation of the fatty acid chain and the subsequent formation of the corresponding aldehydes, carboxylic acids, and high-molecular-weight LPP.

The number of LPP formed over 30 h by induced oxidation (Fenton reaction at 45 °C) were determined. The primary products of the oxidation process are unstable and bound to decompose and lead to homolytic cleavage and the formation of volatile compounds, which are secondary products of oxidation and include aldehydes and ketones. These compounds negatively influence the quality of food products by inducing a rancid odor.

**Figure 1:**
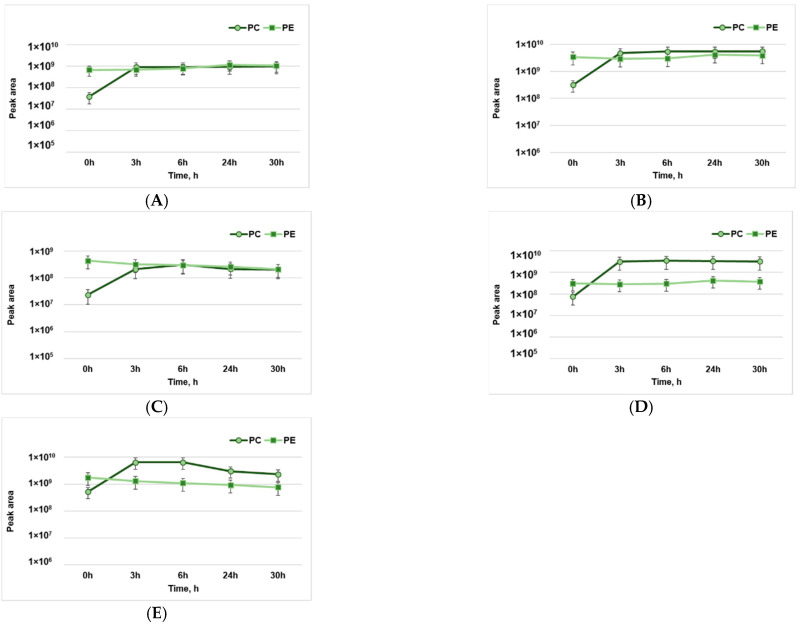
The dynamics of the formation of lipid oxidation products of phosphatidylcholine (PC) and phosphatidylethanolamine (PE): (**A**)—pentanal; (**B**)—hexanal; (**C**)—octenal; (**D**)—hydroxy-nonenal; (**E**)—hydroxy-octadecanal.

In the initial phase of the edible oil oxidation process, triacylglycerols are split and form small concentrations of polyunsaturated fatty acids. These fatty acids serve as a substrate for the initiation of oxidation reactions [25]. In the process of lipid oxidation, hydroperoxides are formed as primary compounds. Peroxides are odorless and tasteless compounds, which, after decomposition, degrade into a variety of secondary products from different classes of compounds such as alkanes, alcohols, esters, aldehydes, and ketones [26]. Even in low concentrations, secondary oxidation compounds such as hexanal, octenal, and decadienal influence the flavor of food products. The oxidation of lipids, especially phospholipids containing unsaturated fatty acids, leads to the formation of a wide range of aldehydic compounds. In this context, the products of phospholipid oxidation were studied (Figure 1).

By analyzing the dynamics of pentanal formation in the process of phospholipid oxidation, we observed a slow change in pentanal formation throughout the 30 h of exposure to pro-oxidant conditions. The initial values of pentanal at 0 h of oxidation for PC lipids are considerably lower compared to those of PE; however, after 3 h, they show a significant increase in the amount of pentanal formed (Figure 1A).

The hexanal formed following the oxidation of PE lipids varied slightly over 30 h. The initial values concerning the oxidation process at 0 h for the PC lipids are considerably lower than those of PE; however, over 3 h, a significant increase in the amount of hexanal content formed could be observed (Figure 1B).

Concerning the evolution of octenal formation in the oxidation process of PE and PC lipids, a considerably higher octenal content in the case of PE lipids was observed compared to the amount of octenal formed by PC lipids. After 3 h of oxidation, an important increase in the amount of octenal was observed for PC lipids, while an unsignificant decrease was observed for PE lipids (Figure 1C).

Hydroxy-nonenal (HNE) is a mutagenic and cytotoxic product of linoleic acid oxidation. The evolution of this compound, which was formed following the oxidation of PE lipids, shows a stable growth throughout the period of exposure to high temperatures (Figure 1D). The increase in HNE content in the case of PC lipids shows a considerable evolution during the first 3 h of exposure to high temperatures. After 24 h of exposure, an unsignificant decrease in HNE content can be seen, which can be explained by the subsequent degradation of aldehydes and the formation of new compounds due to high temperatures.

The formation of hydroxy-octadecanal was also analyzed, for which a significant increase in the number of reaction products formed during the first 3 h of exposure to high temperatures was attested (Figure 1E). Subsequently, the amount of hydroxy-octadecanal gradually decreased over the duration of the experiment. In the case of the reaction products of PE lipids, the highest amount of hydroxy-octadecanal formed was initially observed at 0 h of exposure; then, a gradual decrease in the amount of hydroxy-octadecanal was observed. This fact can be explained by the splitting of hydroxy-octadecanal and the formation of secondary products of the investigated lipid oxidation reaction according to the proposed reaction mechanism [27].

Linoleic acid is an essential precursor of volatile compounds in edible oils and can be easily oxidized to form hexanal, pentanal, heptanal, and *trans*-hept-2-enal [28,29]. Oleic acid is also an important oxidation precursor of volatile compounds, especially nonanal and octanal, which are derived from the oxidative degradation of oleic acid [28]. The oxidative degradation rate of linolenic acid is faster than that of linoleic acid and oleic acid, and linolenic acid is an important source of *trans*-2,4-heptadienal and *trans*-2-hexenal in the oxidation products of edible oils [29]. Volatile compounds exhibit different aroma thresholds, leading to different levels of sensitivity in humans; accordingly, the relative content of these compounds may not reflect their actual contribution to a product’s overall flavor profile.

The autoxidation of linoleic acid involves the removal of the hydrogen from the allylic C11 and the formation of a radical. The radical intermediate reacts with oxygen to produce a mixture of hydroperoxides conjugated at C9 and C13 [30]. The cleavage of hydroperoxide (C13) produces hexanal and pentanal, and the decomposition of the hydroperoxide (C11) of linoleic acid generates heptanal.

Pentanal, hexanal, heptanal, octanal, nonanal, etc., have been identified as key flavor compounds in edible oils. The content of several aldehydes increases with the level of oxidation. In comparison, the content of other aldehydes initially increases and then decreases, possibly because they have been converted to low-molecular-weight compounds in the later stage of oxidation [29].

Although phospholipids are vulnerable molecules, they can act as synergists of some antioxidants, forming effective synergistic antioxidant combinations, for example, with natural tocopherols and carotenoids. The interaction way of synergists and antioxidants is not clearly understood, but it has been shown that tocopherols in the presence of a synergist are depleted at a delayed rate during autoxidation [31].

### 2.2. Characterization of Plant Raw Material

In this section, the individual profiles of carotenoids extracted from sea buckthorn and rose hip fruits will be presented, as this analysis will deepen our understanding of the phenomena observed after their addition with respect to the acquisition of lipophilic extracts used in foodstuffs.

The concentrations of total and individual carotenoids and the antioxidant activity of the dry powder of sea buckthorn were analyzed (Table 2). In the case of rose hip powder, these results were presented in our previous work [10].

The results show that the sea buckthorn dry powder contained a high concentration of carotenoids (66.93 mg/100 g DW). Pop et al. [18] determined the total content of carotenoids in the extracts of six varieties of frozen sea buckthorn, finding that it varied between 53 and 97 mg/100 g DW. Furthermore, Criste et al. [19] analyzed the total content of carotenoids in four varieties of sea buckthorn, demonstrating that the values vary between 35.78 and 5.63 mg/100 g. It was suggested that the variations in the total content of carotenoids in sea buckthorn are dependent on its genetic composition, origin, growing conditions, maturity stage at harvest, storage conditions, and the methods with which it is analyzed.

The identification and quantification of carotenoids in the saponified extracts of sea buckthorn berries were carried out using reversed-phase high-performance liquid chromatography (RP-HPLC). Significant amounts of zeaxanthin (2.54 mg/100 g FW) and all-*trans*-β-carotene (0.45 mg/100 g FW) and small amounts of *cis*-β-carotene (0.14 mg/100 g) FW, γ-carotene (0.12 mg/100 g FW), and lycopene (0.11 mg/100 g FW) were determined in the sea buckthorn extracts. The esters of β-cryptoxanthin, zeaxanthin, and mutaxanthin were not identified in the saponified extracts because they were hydrolyzed, forming considerable amounts of free zeaxanthin, β-cryptoxanthin (0.25 mg/100 g FW), and mutatoxanthin (0.33 mg/100 g FW). Using high-performance liquid chromatography with tandem photodiode array detection (HPLC-PAD), Criste et al. [19] identified five carotenoid compounds, namely, lutein, zeaxanthin, β-cryptoxanthin, *cis-*β-carotene, and β-carotene, in the saponified extracts of four sea buckthorn fruit varieties. Using the HPLC-PAD method for analysis, Pop et al. [18] reported that twenty-seven carotenoid compounds were identified in the unsaponified extracts of six varieties of sea buckthorn, among which were free lutein, zeaxanthin, β-cryptoxanthin, α-carotene, γ-carotene, β-carotene, and lycopene. The carotenoid ester fraction was mainly represented by zeaxanthin esters, followed by lutein and cryptoxanthin esters.

Additionally, the DPPH antioxidant activity was determined in a hydroethanolic extract obtained from sea buckthorn fruits (which was equal to 144.2 TE/100 g FW). Carotenoids are known to be excellent scavengers of singlet oxygen, which can prevent its formation by neutralizing excited sensitizers [32]. Although they insufficiently scavenge free radicals, carotenoids can interact with and neutralize various radical species that can be generated inside cells, except for the peroxyl radical [33]. Wang et al. studied the Chinese plant *Lycium barbarum* L. and found that its flavonoid fraction was more effective in scavenging DPPH free radicals, while the zeaxanthin fraction could more effectively scavenge hydroxyl free radicals [34].

### 2.3. Influence of Temperature on Physicochemical Parameters and Carotenoid Content

Sea buckthorn and rose hip fruits present promising sources of lipophilic biologically active compounds, including carotenoid pigments, which are soluble in organic solvents such as acetone, ethyl ether, chloroform, ethyl acetate, petroleum ether, hexane, etc. [35]. Nevertheless, these solvents cannot be used in the food industry because of their toxicity; therefore, refined and deodorized sunflower oil was proposed as a solvent for the extraction of carotenoids, containing a high concentration of mono- and polyunsaturated acids [36]. The aim of this research was to determine the influence of temperature on the extraction yields of carotenoids in lipophilic extracts obtained from sea buckthorn and rose hips. At the same time, the quality parameters and stability of the lipophilic extracts obtained from the berries during storage were analyzed.

The physicochemical quality parameters of the lipophilic extracts of sea buckthorn and rose hips obtained at different extraction temperatures were compared with those of sunflower oil, which was used as an extractant (Table 3).

It was confirmed that the physicochemical quality parameters of the sea buckthorn and rose hip lipophilic extracts based on sunflower vegetable oil vary but remain within the permissible limits for refined and deodorized edible sunflower oil (acid value—max. 0.4 mg KOH/g and peroxide value—max. 10 mmol O_2_/kg) [37].

The conditions for obtaining lipophilic extracts from sea buckthorn and rose hips at different temperatures were investigated to ensure a maximum yield of carotenoids. The variation in temperature from 30 °C to 65 °C changed the extraction yields of lipophilic carotenoids from sea buckthorn and rose hip berries, demonstrating that the highest level of pigments was reached at 45 °C, which then decreased at 65 °C. In the berry extracts, zeaxanthin and lycopene had the highest yields: for sea buckthorn—9.55 mg/100 g DW and 9.40 mg/100 g DW, and for rose hip—24.36 mg/100 g DW and 24.01 mg/100 g DW, respectively. Lutein was only detected in the sea buckthorn extracts, which was also demonstrated by HPLC analysis, for which the determined content was 7.76 mg/100 g DW at 45 °C and 6.84 mg/100 g DW at 65 °C [37].

Sea buckthorn berries are known for their high content of tocochromanol (all four tocopherols and tocotrienols isomers), which are present in both the pulp and seeds of the berries. According to Kallio et al., the seeds of the subspecies *rhamnoides* have an average total tocochromanol content of 290 mg/kg, while the fruit flesh contains an average 40 mg/kg [38]. Andersson et al. found a total tocochromanol content ranging from 400–800 µg/g in the lyophilized whole berries of *Hippophae rhamnoides*, for which there were large variations depending on the cultivar, harvest time (ripening stage), and year [39]. Regardless of the subspecies and cultivar, α-tocopherol was the major compound, with more than 80% of the total tocochromanol content in the soft parts of the berries and up to 50% in the seeds [38].

Rose hips (*Rosa canina* L.) are berries with high content of lipophilic antioxidants, both carotenoids and tocopherols. In ripened hips from Portugal, the total tocopherol content was around 80 mg/100 g at dry weight (DW), while in the ripened seeds this value was only 1.70 mg/100 g DW [40]. Investigations concerning various Rosa species form Sweden revealed a total tocopherol fraction ranging between 116.7 to 248.1 µg/g DW [38].

Tocopherols and tocotrienols are strong lipophilic antioxidants, which can act syn-eristically with carotenoids in biological systems, preventing, in particular, the oxidation of unsaturated fatty acids [41]. Besides carotenoids, tocopherols from sea buckthorn and rose hips could induce a positive effect on the antioxidant activity of the oil. However, sunflower oil has a much higher α-tocopherol content than berries (an average of 40 mg/100 g oil). Considering the ratio between berries and sunflower oil used for extraction and the small contribution of tocopherols from berries to the total amount of tocopherols in lipophilic extracts, we can assume that the improved oxidative stability of SLE and RLE can be attributed to carotenoids. Although both berries contain other antioxidants, such as phenolic compounds and ascorbic acid, these are not extractable with vegetable oil due to their polar and hydrophilic characters.

The reduction in the carotenoid extraction yield at 65 °C is explained by the fact that the thermal treatment caused the formation of different *cis* isomers of carotenoids, thus reducing the content of *trans* carotenoids. In addition, carotenoids have a different capacity to form cis isomers. All-*trans-*β-carotene can be easily isomerized to the *cis* configuration when exposed to heat. Isomerization energy is involved in relocating the single or double bond from one form of a carotene to another [42]. Regarding the effect of thermal processing, 13-*cis-*β-carotene is the main product of geometric isomerization [43]. Lycopene contains eleven conjugated double bonds and can be isomerized in mono-*cis* or poly-*cis* configurations, among which 5-*cis*-lycopene is the most stable isomer, followed by all-*trans*- and 9-*cis*-lycopene. Additionally, 5-*cis*-lycopene has the lowest isomerization energy among the other *cis*-lycopene isomers [44].

In sunflower oil, which is rich in polyunsaturated acids, the degradation of carotenoids increases due to the faster oxidation of unsaturated lipids, which produce radicals that are likely to attack carotenoids [45]. It was found that when the extraction temperature increased from 45 to 65 °C, the individual content of the carotenoids in the sea buckthorn extract decreased from 8.45 mg/100 g DW to 7.47 mg/100 g DW (β-carotene), from 9.40 mg/100 g DW to 8.31 mg/100 g DW (lycopene), from 9.55 mg/100 g DW to 8.44 mg/100 g DW (zeaxanthin), from 8.87 mg/100 g DW to 7.80 mg/100 g DW (β-cryptoxanthin), and from 7.76 mg/100 g DW to 6.84 mg/100 g DW, respectively (lutein). The same trends were found in the case of rose hip extract. Carotenoids can be effective antioxidants due to their delocalized electrons, which can be stabilized by reactive resonance intermediates such as carbocations or radicals. They can neutralize singlet oxygen and eliminate active free radicals that are involved in the process of lipid peroxidation. The singlet oxygen scavenging activity of a carotenoid depends strongly on the number of conjugated double bonds present in the structure of the molecule and to a lesser degree on the carotenoid groups (cyclic or acyclic) present at the end of the chain or on the nature of the carotenoid substituents containing cyclic groups [46].

UV–Vis absorption spectra of the lipophilic extracts of berries and sunflower oil were analyzed as a function of extraction temperature (Figure S1). The π electrons of the conjugated double-bond system in carotenoids are delocalized, and the excited state, having a relatively low energy, corresponds to light in the visible region in the wavelength range from 400–500 nm, which causes their color. The transition involved corresponds to π → π*, where one of the π bond electrons in the conjugated double bond system migrates to a free π* orbital. The conjugated double-bond system is a chromophore that gives carotenoids their color, providing the visible absorption spectra and serving as the basis for pigment identification and quantification [33]. Carotenoid pigments in the spectra of sea buckthorn fruit extracts present three important absorption bands in the visible range: the first at λ_max_ = 435–437 nm; the second at λ_max_ = 458–460 nm; and the third at λ_max_ = 480–484 nm. In the case of rose hip extracts, two essential bands are presented in the spectra: the first at λ_max_ = 459–460 nm and the second at λ_max_ = 479–481 nm. The absence of an absorption maximum in the spectra of sunflower oil attests the lack of carotenoid pigments due to the application of refining and deodorization operations applied in the process of obtaining the oil, with the pigments being thermally decomposed and removed [43].

FTIR spectra of lipophilic extracts of sea buckthorn and rose hips were also analyzed as a function of extraction temperatures (Figure S2). It was found that the lipophilic extracts absorb radiant energy at two specific wavelengths in the FTIR range (λ_max_ = 3.45 μm and 5.73 μm) and two specific wavelengths in the near FTIR range (1724 cm^−1^ and 1230 cm^−1^). The symmetric, asymmetric (ν), or deformation (δ) vibration of the characteristic groups of lipids at these wavelengths causes the variation in absorption, which is directly correlated to the content of fats, carriers of these groups [44]. The spectra were analyzed according to two wavelengths specific to lipids: 1748 cm^−1^ for the C=O carbonyl group of unsaturated acids, and 1164 cm^−1^ (resonance band) with two harmonic bands at 1100 cm^−1^ and 1296 cm^−1^, which are specific for the group C–O. It was established that, practically speaking, the intensity of the light absorption bands at these wavelengths does not vary, regardless of the extraction time, and that they correspond to the bibliographic data for sunflower oil [45]. In the 3100–2800 cm^−1^ region, C–H valence oscillations were recorded for the saturated carbon atoms, namely, CH_3_—2988 cm^−1^ and CH_2_—2852 cm^−1^, the aliphatic groups from the alkyl residue of triglycerides found in large quantities in vegetable oils. These groups also form absorption bands in the 1466 cm^−1^ and 1378 cm^−1^ regions, respectively. The presence of the double bond in the compounds can be demonstrated by the ν (=CH) band in the 3015 cm^−1^ region. The deformation oscillations of this =CH group are located in the 650 cm^−1^–750 cm^−1^ region, where an important maximum was recorded. The extraction temperature did not influence the position of the specific absorption maxima. However, with the increase in the extraction temperature, a greater intensity of the spectral lines characteristic of double bonds is attested. This fact demonstrates the favorable influence of the extracted antioxidants on the oxidative stability of sunflower oil.

### 2.4. Influence of Storage Time on the Formation of Lipid Oxidation Products and Carotenoid Content

The dynamics of oxidation largely depend on the composition of fatty acids and the content and activity of antioxidants and prooxidants (air, heat, light, etc.). The autoxidation of sunflower oil begins with the involvement of esterified unsaturated fatty acids in phospholipids, which form hydroperoxides, for which the simultaneous release of energy is transferred to the other molecules they activate. This process continues until an antioxidant breaks the autoxidation chain. The antioxidant can act either by consuming oxygen or consuming the energy released by the first molecules that react with oxygen. Regardless of the mode in which the antioxidant functions, it cannot prevent oxidation indefinitely because it is itself consumed in the process of lipid protection [46].

Chemical and enzymatic oxidation can occur, but the lipoxygenases (which are responsible for enzymatic oxidation) present in sunflower oil and berries are inactivated by heating during refinement and drying [47]. For this reason, it is important to investigate the evolution of the physicochemical properties of the lipophilic extracts obtained at 45 °C during storage for 12 months at 4 ± 1 °C in the absence of light. The physicochemical parameters describing the oxidative states of the oil and berry extracts during storage are presented in Table 4.

The acid value (AV) indicates the extent of the heat-induced oxidative and hydrolytic degradation of the oil and lipophilic extracts of sea buckthorn and rose hips. The AV values in the lipophilic extracts at the acquisition stage vary within 0.20–0.21 mg KOH/g, which are higher in relation to the values for sunflower oil, namely, 0.17 mg KOH/g. This fact is due to the presence of lipids in the pulp of sea buckthorn and rose hips, which are essential components of the cell membrane, thereby ensuring the functioning of receptors, enzymes, ion channels, and other substance transport systems in the pulp matrix [47]. During the storage of oil and lipophilic extracts, the AV value increased, demonstrating the accumulation of free fatty acids without exceeding the limits set by regulations (max. 0.4 mg KOH/g oil).

The peroxide value (PV) determines the degree of stability of the extracts. During storage, the sea buckthorn extracts’ PV increased: in sea buckthorn, the increase was within the range of 1.31 to 3.24 mmol O_2_/kg; in rose hips, the range was 1.62–3.71 mmol O_2_/kg; and in oil, the increase ranged from 2.82 to 5.12 mmol O_2_/kg. The PV values in the lipophilic extracts are lower compared to sunflower oil due to the presence of carotenoids, i.e., antioxidants extracted from the pulp of berries, which can neutralize singlet oxygen and eliminate active free radicals involved in the lipid peroxidation process [48]. Thus, the susceptibility of polyunsaturated fatty acids to oxidation is modulated, the nutritional value of the extracts is maintained, and their storage quality increases. Delgado-Vargas et al. [49] studied chlorophylls sensitized to the photooxidation of soybean oil in the presence of carotenoids (lutein, zeaxanthin, and lycopene), finding that carotenoid pigments contributed to the decrease in the PV by neutralizing singlet oxygen. The efficiency of sea buckthorn and rose hip antioxidants in lipophilic extracts depends not only on their structural characteristics (their chemical reactivity towards peroxyl and other active components) but also on many other factors, such as their storage temperature, the presence of light, antioxidant concentrations, the type of substrate involved, the physical state of the system, and numerous micro-components, which can act as prooxidants or synergists [50]. The PV values of all the investigated samples during storage did not exceed regulatory limits (max. 10 mmol O_2_/kg).

Conjugated dienes and trienes represent indicators with which to measure the oxidative state of oils, which are produced from unsaturated fatty acids as a result of the rearrangement of double bonds. These products show absorption maxima at different wavelengths: conjugated dienes—at 236 nm and conjugated trienes—at 273 nm. Our analysis of the obtained data demonstrates an increase in the content of conjugated dienes and trienes in all the investigated samples during the 12-month storage period. The minimum content was found in sea buckthorn extracts, varying between 7.20–10.95 µmol/g for conjugated dienes and 3.34–5.09 µmol/g for conjugated trienes; in rose hip extracts, a variation in the range of 7.25–11.31 µmol/g for dienes and 3.37–5.33 µmol/g for trienes was observed, while in sunflower oil the values concerning the evolution of conjugated dienes and trienes were 8.60–13.66 µmol/g and 3.99–6.35 µmol/g, respectively. The reduced formation dynamics of the conjugated dienes and trienes in the lipophilic extracts is obviously due to the beneficial influence of some compounds from sea buckthorn and rose hips on the complex of unsaturated fatty acids from triglycerides.

Unlike the PV, the value of *p*-anisidine measures the number of secondary oxidation products, such as carbonyl compounds (aldehydes, ketones, and their derivatives) with different carbonyl chain lengths, which can negatively affect taste and odor. An insignificant increase in the *p*-anisidine value during storage was established in the oil and extracts of sea buckthorn and rose hips. During storage, the lowest number of secondary oxidation products accumulated in the berry lipophilic extracts, for which the values of the *p*-anisidine value varied within the range from 0.986 to 0.991 c.u.

Analysis of the changes in the physicochemical properties of the investigated samples during storage for 12 months at a temperature of 4 °C and in the absence of light showed that under these conditions, the carotenoids from the sea buckthorn and rose hip extracts positively influenced the oxidative stability of the extracts in relation to sunflower oil. Crapiste et al. [51] studied the effect of temperature on the oxidation of sunflower oils during storage. They demonstrated that the rate of lipid oxidation strongly depends on the presence of oxygen and the storage temperature, noting that a low temperature and limited oxygen availability reduced the oxidation rate, thus increasing oil stability.

Furthermore, within the framework of this research, the content of the individual carotenoids in the lipophilic extracts of sea buckthorn and rose hips was monitored during the 12-month storage period (Table 4).

Based on the obtained data, it was established that during the 12 months of storage at 4 ± 1 °C in the sea buckthorn and rose hip extracts, the content of individual carotenoids decreased. The concentration of β-Carotene decreased from 8.45 to 6.51 mg/100 g DW (SLE) and from 21.56 to 16.26 mg/100 g DW (RLE); that of lycopene decreased from 9.40 to 6.41 mg/100 g DW (SLE) and from 24.01 to 16.43 mg/100 g DW (RLE); that of zeaxanthin decreased from 9.55 to 7.35 mg/100 g DW (SLE) and 24.36 to 18.38 mg/100 g DW (RLE); that of β-cryptoxanthin decreased from 8.87 to 6.26 mg/100 g DW (SLE) and 22.68 to 14.96 (RLE); and that of lutein decreased from 7.76 to 5.29 mg/100 g DW (SLE).

The reduction in the carotenoid content of the lipophilic extracts during storage was most likely due to geometric isomerization and oxidation. The degree of isomerization is directly correlated with the intensity and duration of the heat treatment applied. Initially, some of the all-*trans*-carotenoids are isomerized in the *cis* configuration, and both the *cis* and *trans* configurations are subjected to the oxidation process [33]. The oxidation of carotenoids begins with epoxidation and cleavage to apocarotenals. The detection of epoxycarotenoids and apocarotenals with hydroxyl groups indicates that the hydroxylation reaction is also involved [33]. Subsequent fragmentations generate compounds with low molecular weight (similar to those formed during the oxidation of fatty acids). Cleavage, which can occur at various positions in the polyene chain, directly produces short, volatile fragments. Volatile compounds are mainly represented by aldehydes, ketones, alcohols, and hydrocarbons, which contribute to the formation of unpleasant odors in food.

Qiu et al. [52] investigated the stability of tomato puree that was stored at 4 °C and 24 °C, demonstrating that increasing the storage temperature led to a total loss of lycopene and to an increase in the percentage of isomerized lycopene. The greatest stability of lycopene was observed at 4 °C. In the case of tomato juice, it was shown that during storage at 4 °C, lycopene decreased exponentially in both treated and untreated samples [53]. Schweiggert et al. [54] found that during storage for 4 months at ambient temperature in the presence but also in the absence of light, the carotenoid content decreased by 17% and 10% in chili powder and by 40% and 39% in paprika powder, respectively.

The antioxidant activity of carotenoids is a direct consequence of their long polyene chain, which is a highly reactive, electron-rich system of conjugated double bonds that are susceptible to attack by electrophilic reagents and form stabilized radicals. This structural characteristic is mainly responsible for the chemical reactivity of carotenoids towards oxidizing agents and free radicals [33]. According to Kiokias et al., carotenoids deactivate singlet molecular oxygen. Singlet oxygen will preferentially transfer exchange energy to produce carotene in the triplet state, while oxygen returns to its ground energy state. A carotenoid in the triplet state releases energy as heat and returns to its normal energy state. Thus, carotenoids act so effectively that one carotenoid molecule is capable of quenching about 1000 singlet oxygen molecules [31].

### 2.5. Mathematical Modeling of the Oxidation Processes of Sunflower Oil Stabilized with Sea Buckthorn and Rose Hips Extracts

**Figure 2:**
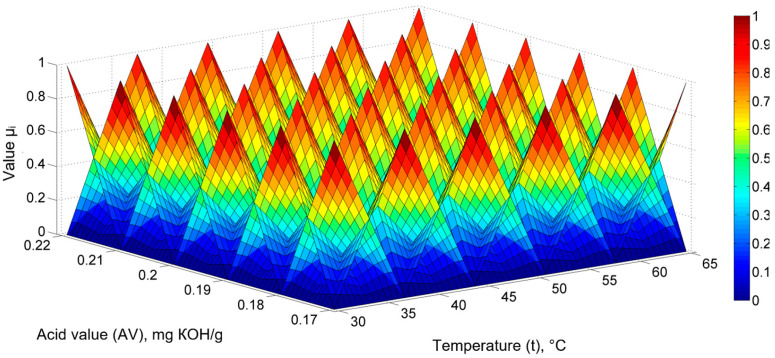
Generalized fuzzy mathematical model PV = f (t, AV) for sunflower oil, sea buckthorn, and rose hip extracts: triangular fuzzy set representation (6 and 6).

The data highlight the existence of more or less pronounced nonlinear dependencies between the quantities, thus presenting implications for mathematical models. The existence of some dependencies between the physicochemical parameters also allows for the establishment of mathematical models focusing on both temperature and said parameters. A generalized mathematical model was established for sunflower oil, sea buckthorn, and rose hip extracts of the type PV = f (t, AV), thereby providing the PV values as a function of the extraction temperature and the AV using fuzzy sets. As two factorial quantities, spatial, triangular fuzzy sets were adopted, as can be seen from Figure 2, whose right side depicts a graduated scale concerning values and colors with the value *μ_i_*.

To establish a mathematical model where PV = f (t, AV), six fuzzy sets were adopted along the temperature axis and six sets were adopted along the AV axis, resulting in a total of 36 sets; consequently, the fuzzy model has 36 coefficients (*θ*) [55].

**Figure 3:**
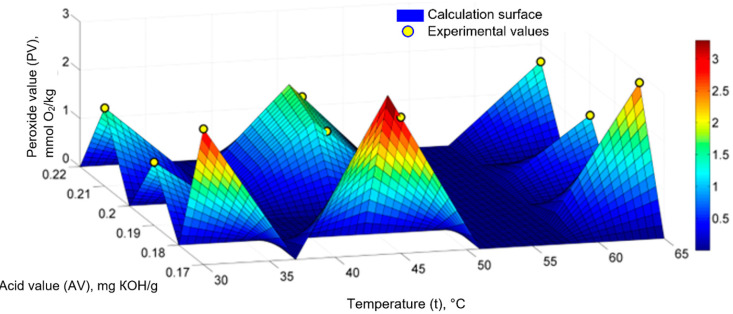
Generalized fuzzy mathematical model (PV = f (t, AV)) for sunflower oil, sea buckthorn, and rose hip extracts: experimental values and computational surface.

Figure 3 shows the fuzzy calculation surface, on which the nine points with the experimental values of the PV values for the sunflower oil, sea buckthorn, and rose hip extracts are arranged. The weights of the fuzzy sets were determined based on the values of 36 *θi* coefficients of the fuzzy model and the experimental values.

The graphs in Figure S3 contain the weights of the fuzzy sets *βi* (A), the values of the 36 coefficients *θi* of the fuzzy model (B), and the experimental values and those of the fuzzy model (C). Mathematical models based on fuzzy set indicate the existence of diversified phenomena between influencing factors and measured parameters. Mathematical modeling based on fuzzy sets was applied to research the influence of the concentration of ethyl alcohol on the extraction of the content of biologically active compounds from berry and grape marc [55].

**Figure 4:**
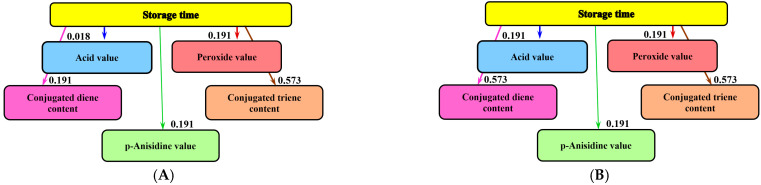
Informational analysis regarding the influence of storage time on the physicochemical quality indices of the lipophilic extracts: (**A**) sea buckthorn; (**B**) rose hip.

Informational analysis was used to establish the influence of storage time on the physicochemical quality parameters of the lipophilic extracts of sea buckthorn and rose hips (Figure 4). This mathematical-modeling method was applied to study the influence of days of storage on the quality of yogurt with apple pomace (20 days) [56] and dry aged beef (35 days) [57]. It was also used to analyze the influence of different amounts of vegetal addition on the quality of bread and gingerbread [10,20].

With regard to the liposoluble extracts of sea buckthorn and rosehip, storage time has the greatest influence on the content of conjugated trienes (mutual information: 0.573 bits). The influence on conjugated dienes was lower in the case of sea buckthorn extract: 0.191 bits. For all the investigated extracts, a reduced influence on AV (0.018 bits and 0.191 bits), *p*-anisidine, and PV (0.191 bits for all investigated samples) was highlighted.

The mutual information analysis demonstrated that carotenoids from berries can prevent the oxidation of lipids within 12 months, as they are themselves oxidized in the process of lipid protection. However, throughout this protective period, smaller amounts of hydroperoxides are formed. In this way, the storage period of the oils is extended, during which no unpleasant flavors will be detected.

## 3. Materials and Methods

### 3.1. Chemical Materials

Reagents of chromatographic purity obtained from Sigma-Aldrich (Merck KGaA. Darmstadt, Germany) and ethyl alcohol of LC-MS-grade obtained from Carl Roth (Karlsruhe, Germany) were used for this research. DPPH (2,2-diphenyl-1-picrylhydrazyl), copper (II) sulfate pentahydrate, Na-l-Ascorbate, *p*-Anisidine, potassium hydroxide, ethyl acetate, methanol, glacial acetic acid, and potassium iodide were provided by Sigma-Aldrich (Merck KGaA. Darmstadt, Germany). Carotenoid standards (β-carotene, lycopene, β-cryptoxanthin, lutein, and zeaxanthin) were purchased from Extrasynthese (Genay, France). All spectrophotometric measurements were performed on an Analytik Jena Specord 200 Plus (Jena, Germany) spectrophotometer.

### 3.2. Biological Material

Sea buckthorn fruit (*Hippophae rhamnoides*) of the “Clara” variety was harvested from a plantation in the village of Pohrebea (Republic of Moldova) [20]. Rose hip (*Rosa canina* L.) fruit of the “VisocoVitaminnii” variety was harvested from a plantation in the village of Taul (Republic of Moldova) [10]. Sea buckthorn fruit and seedless rose hip pulp were dried at 65 ± 1 °C to a moisture content of 6.8 ± 0.4%, crushed to powder, and sieved. Refined and deodorized sunflower oil “Floris” (Balti, Republic of Moldova) was used to obtain the lipophilic extracts. For the analysis of the products and the kinetics of forced autoxidation, unrefined vegetable oil that had been cold-pressed from sunflower seeds and purchased at the local market (Republic of Moldova) was used, which corresponded to the quality and harmless conditions for this category (oil) [37].

### 3.3. Analysis of Lipid Oxidation Products

The identification of lipid oxidation products was performed using LC-MS/MS tandem mass spectrometry. Phosphatidylcholine and phosphatidylethanolamine served as standards [58]. In order to obtain lipid oxidation products, the forced oxidation process was simulated through the Fenton mechanism. CuSO_4_·5H_2_O (1.2 mmol/L; 250 µL) and Na-l-Ascorbate (2.4 mmol/L; 250 µL) were added to the analyzed lipid samples [59,60,61,62].

Separation of LPPs from oxidized lipids was performed via reversed-phase liquid chromatography on a C18 column. Evaluation of plasmalogens oxoPE and oxoPC was performed with both ESI ionization in negative ion mode and ESI ionization in positive ion mode. Samples were analyzed by direct infusion using Nanospray emitters on an ESI-LTQ-Orbitrap analyzer operating in positive ion mode. MS spectra were recorded in Fourier-transform-mass-spectrometry-scanning mode with a target mass resolution of 100,00 at *m*/*z* 400. An isolation width of 1–1.5 µ was used in CID fragmentation experiments [63,64]. The recorded spectra were analyzed using the XCalibur 4.2 software application (Thermo Fisher Scientific Inc., Waltham, MA, USA).

### 3.4. Carotenoid Extraction

The carotenoid extract of sea buckthorn was obtained by the method described by Ghendov-Mosanu et al. [20]. The extract was saponified with 30% methanolic KOH, washed, and purified. The carotenoid-containing supernatant was dried and evaporated to dryness. Samples for analysis were dissolved in ethyl acetate and filtered through 0.45 mm polytetrafluoroethylene filters. The obtained samples were analyzed by RP-HPLC [65]. All extractions were performed in triplicate.

### 3.5. Separation of Carotenoids by RP-HPLC

Individual carotenoid content was determined using RP-HPLC, which was performed using a Shimadzu LC-20AT with an SPD-M20A diode array detector (DAD) (Shimadzu Corporation, Kyoto, Japan), as described by Ghendov-Mosanu et al. [10]. Comparison of the UV–Vis spectra and the retention times of the sample peaks with those of the standard solutions and spectral data reported in the literature allowed for the identification of carotenoids from sea buckthorn samples (Table 5).

### 3.6. Antioxidant Activity by Reaction with DPPH Radical

The sea buckthorn samples were used to prepare 60% hydroethanolic extracts at temperature of 65 °C. The solid/liquid ratio was 1:12 (*w*/*v*) [66], and mechanical stirring was conducted at 60 rpm for 90 min. The obtained extract was filtered and stored in glass bottles at temperature of 4.0 ± 1.0 °C. The method described by Brand-Williams et al. [67] was also used to determine antioxidant activity. The results were expressed as mmol TE/100g DW from a calibration curve (0–250 μmol/L) with trolox.

### 3.7. Lipophilic Extract Characterization

Lipophilic extracts were obtained from the sea buckthorn (SLE) and rose hip (RLE) powders and refined and deodorized sunflower oil in ratios of 1:12 (*w*/*v*) and 1:14 (*w*/*v*), respectively [66], in a water bath at temperatures of 30 °C, 45 °C, and 65 °C; under mechanical stirring at 60 rpm for 90 min; and under limited light penetration conditions. The lipophilic extracts were centrifuged at 8000 rpm for 10 min, filtered, and stored in glass bottles at 4 ± 1 °C. The refined and deodorized sunflower oil was used as a control sample and treated under the same conditions.

#### 3.7.1. Physicochemical Analysis of Sunflower Oil and Lipophilic Extracts

The acid value, peroxide value, conjugated dienes and trienes, and *p*-anisidine values of the sunflower oil and lipophilic extracts were determined according to the following methods: AOCS Official Method Cd 3d-63 1999 [68], AOCS Official Method 8-53 2003 [69], AOCS Official Method Cd 18-90 2017 [70], and AOCS Official Method Cd 7-58 (Revised 2022) [71]. Determinations were performed initially and then after 3, 6, and 12 months of storage.

#### 3.7.2. UV–Vis and FTIR Analysis of Oil and Lipophilic Extracts

UV–Vis spectra of the sunflower oil and the lipophilic extracts were recorded in the 330–630 nm range using Analytik Jena Specord 200 Plus spectrophotometer (Jena, Germany).

The sunflower oil and the lipophilic extracts were characterized by Fourier transform infrared spectroscopy (FTIR) for the qualitative evaluation of functional groups. An FTIR-4000 Series Spectrometer (Jasco, Japan) was used to obtain the infrared spectra recorded in the range from 400 to 4000 cm^−1^. Spectra were recorded at the initial stage of sample storage.

#### 3.7.3. Total Carotenoid Content

Total carotenoid content was determined using the spectrophotometric method [72]. For the lipophilic extracts, deodorized refined oil was used as a control sample. For each analyzed sample, absorbance was determined at following the wavelengths: 448 nm, 452 nm, and 470 nm [72,73]. Determinations were performed initially and after 3, 6, and 12 months of storage.

### 3.8. Mathematical Modeling

Mathematical modeling (using fuzzy sets and analysis of mutual information of experimental data) was performed using the MATLAB program (MathWorks, Inc., Natick, MA, USA). To establish a generalized mathematical model for sunflower oil, sea buckthorn, and rose hip lipophilic extracts of the type PV = f (t, AV), the PV values were used as a function of the extraction temperature and the AV values by applying fuzzy sets [55].

Analysis of mutual information was used to determine the influence of the storage time on the physicochemical quality parameters of sunflower oil, sea buckthorn, and rose hip lipophilic extracts. Names of physicochemical quality parameters are shown in the rectangles of corresponding the graph. Bits are units measuring mutual information values, which are indicated on the graph arrows. The more pronounced the influence of storage time on the physicochemical quality parameters, the higher the bit value [74].

### 3.9. Statistical Analysis

All calculations were performed using Microsoft Office Excel 2007 (Microsoft, Redmond, WA, USA). Data obtained in this study are presented as mean values ± the standard error of the mean, which were calculated for three parallel experiments. Comparison of average values was based on one-way analysis of variance (ANOVA), which was conducted according to Tukey’s test and with results presented at a significance level of *p* ≤ 0.05, using the Staturphics program, Centurion XVI 16.1.17 (Statgraphics Technologies, Inc., The Plains, VA, USA).

## 4. Conclusions

The lipid oxidation metabolites of sunflower oil were analyzed via LC-MS/MS using electrospray ionization (ESI) in negative and positive modes. The evaluation of the plasmalogens oxPE and oxPC was performed in both polarization modes. A series of lipid oxidation products was identified, with the dominant ones being hexanal (*m*/*z* = 98.2021), octenal (*m*/*z* = 124.1121), hydroxynonenal (*m*/*z* = 154.2390), and hydroxyoctadecanal (*m*/*z* = 282.2792). The applied method allowed for the determination of the chemical changes that occur during lipid oxidation and the confirmation of the lipid oxidation mechanisms.

The individual profiles of the carotenoids from sea buckthorn were determined using RP-HPLC, which identified zeaxanthin and all-*trans-*β-carotene as the major carotenoids, while smaller amounts of *cis-*β-carotene, γ-carotene, and lycopene were identified. The DPPH antioxidant activity observed was 144.2 ± 0.4 mmol TE/100 g FW. The increase in temperature from 30 °C to 45 °C had a positive effect on the extraction yield of carotenoids from berries, but the further increase to 65 °C negatively affected the total and individual carotenoid content.

The dynamics of the accumulation of the primary and secondary products of lipid oxidation and the variation in the carotenoid pigment content in the lipophilic extracts of sea buckthorn and rose hips during storage demonstrated good stability at 4 °C in the absence of light for 12 months.

A generalized mathematical model was established for sunflower oil, sea buckthorn, and rose hip extracts of the type PV = f (t, AV) using fuzzy sets. Mutual information analysis was applied to determine the influence of storage time on the physicochemical quality of the sea buckthorn and rose hip lipophilic extracts.

## Figures and Tables

**Table 1:** Products of lipid oxidation.

Aldehyde	Molar Mass, g/mol	Chemical Formula	Formation Path	RT
Pentanal	86.13	C_5_H_10_O	Truncation	3.11
Hexanal	100.16	C_6_H_12_O	Truncation	3.73
Octenal	126.20	C_8_H_14_O	Truncation	5.09
Hydroxynonenal	156.11	C_9_H_16_O_2_	Truncation	3.73
Hydroxy Octadecanal	284.48	C_18_H_36_O_2_	Truncation OH addition	12.59
PC (1:0/20:4 <oxo@C1>)	571.68	C_29_H_50_NO_8_P	Truncation	25.52
PC (P-18:1/5:0 <oxo@C5>)	605.78	C_31_H_60_NO_8_P	Truncation	11.19
PC (P-18:0/11:2 <OH@C5>)	659.83	C_34_H_62_NO_9_P	Truncation OH addition	11.61
1-LysoPC	543.67	C_28_H_50_NO_7_P	Truncation	4.75
PC (P-18:0/20:4 <epoxy@sn2>)	810.13	C_46_H_84_NO_8_P	Epoxidation	16.75
PC (P-18:0/20:4 <OH@C11>)	810.13	C_46_H_84_NO_8_P	OH addition	17.21
PC (P-18:0/7:1 <oxo@C7>)	631.82	C_33_H_62_NO_8_P	Truncation	10.14

**Table 2:** The individual carotenoids and the antioxidant activity of sea buckthorn fruit (the results are expressed as means ± standard deviations of three experiments).

Indices	Quantity
Mutatoxanthin, mg/100 g fresh weight (FW)	0.33 ± 0.06
Lutein, mg/100 g FW	0.38 ± 0.03
Zeaxanthin, mg/100 g FW	2.54 ± 0.08
β-Cryptoxanthin, mg/100 g FW	0.25 ± 0.04
*cis*-β-Carotene, mg/100 g FW	0.14 ± 0.02
all-*trans*-β-Carotene mg/100 g FW	0.45 ± 0.05
γ-Carotene, mg/100 g FW	0.12 ± 0.01
Lycopene, mg/100 g FW	0.11 ± 0.01
DPPH Antioxidant activity, mmol trolox equivalent (TE)/100 g FW	144.2 ± 0.4

DPPH = 2,2-diphenyl-1-picrylhydrazyl-hydrate.

**Table 3:** The influence of temperature on the physicochemical parameters and carotenoid content of the sea buckthorn and rose hip lipophilic extracts and sunflower oil.

Parameter	Sunflower Oil			SLE			RLE		
	30 °C	45 °C	65 °C	30 °C	45 °C	65 °C	30 °C	45 °C	65 °C
Acid value, mg KOH/g	0.17 ± 0.01 ^a,b^	0.17 ± 0.01 ^a,b^	0.18 ± 0.01 ^b,c^	0.19 ± 0.01 ^c,d^	0.20 ± 0.0 ^d^	0.20 ± 0.0 ^d^	0.21 ± 0.01 ^e,f^	0.21 ± 0.01 ^e,f^	0.22 ± 0.0 ^f^
Peroxide value, mmol O_2_/kg	2.81 ± 0.01 ^c^	2.82 ± 0.0 ^c^	2.82 ± 0.0 ^c^	1.31 ± 0.02 ^a^	1.31 ± 0.02 ^a^	1.33 ± 0.01 ^a^	1.62 ± 0.01 ^b^	1.62 ± 0.01 ^b^	1.63 ± 0.01 ^b^
Content of conjugated dienes, µmol/g	8.59 ± 0.05 ^c^	8.60 ± 0.06 ^c^	8.66 ± 0.05 ^c^	7.18 ± 0.06 ^a^	7.20 ± 0.05 ^a^	7.29 ± 0.06 ^a,b^	7.23 ± 0.04 ^a^	7.25 ± 0.06 ^a,b^	7.32 ± 0.05 ^b^
Conjugated triene content, µmol/g	3.97 ± 0.02 ^b^	3.99 ± 0.03 ^b,c^	4.01 ± 0.04 ^b,c^	3.32 ± 0.02 ^a^	3.34 ± 0.03 ^a^	3.37 ± 0.02 ^a^	3.36 ± 0.02 ^a^	3.37 ± 0.03 ^a^	3.40 ± 0.0 ^a^
*p*-anisidine value, u.c.	0.848 ± 0.001 ^e,f^	0.848 ± 0.001^e,f^	0.849 ± 0.0 ^f^	0.841 ± 0.001 ^a,b^	0.841 ± 0.001 ^a,b^	0.842 ± 0.0 ^b^	0.844 ± 0.001 ^c,d^	0.844 ± 0.001 ^c,d^	0.845 ± 0.0 ^d^
β-Carotene, mg/100 g DW	nd	nd	nd	7.58 ± 0.02 ^a^	8.45 ± 0.05 ^b^	7.47 ± 0.09 ^a^	20.59 ± 0.18 ^c^	21.56 ± 0.15 ^c^	19.70 ± 0.11 ^c^
Lycopene, mg/100 g DW	nd	nd	nd	8.42 ± 0.08 ^a^	9.40 ± 0.09 ^b^	8.31 ± 0.08 ^a^	22.88 ± 0.12 ^d^	24.01 ± 0.17 ^d^	21.94 ± 0.16 ^c^
Zeaxanthin, mg/100 g DW	nd	nd	nd	8.57 ± 0.07 ^a^	9.55 ± 0.09 ^a,b^	8.44 ± 0.09 ^a^	23.26 ± 0.15 ^c^	24.36 ± 0.14 ^c^	22.26 ± 0.19 ^c^
β-Cryptoxanthin, mg/100 g DW	nd	nd	nd	7.95 ± 0.05 ^a^	8.87 ± 0.09 ^a^	7.80 ± 0.08 ^a^	21.65 ± 0.09 ^b^	22.68 ± 0.07 ^b^	20.75 ± 0.09 ^b^
Lutein, mg/100 g DW	nd	nd	nd	6.95 ± 0.03 ^a,b^	7.76 ± 0.05 ^c^	6.84 ± 0.06 ^a^	nd	nd	nd

nd = not detected; SLE = sea buckthorn lipophilic extract; RLE = rose hip lipophilic extract. Results are presented as mean value ± standard deviation. Different letters (^a–f^) designate statistically different results (*p* ≤ 0.05).

**Table 4:** Evolution of the physicochemical parameters and carotenoid content of the sea buckthorn and rose hip lipophilic extracts and sunflower oil during storage.

Parameter	Research Period	Name of the Sample		
		Sunflower Oil	SLE	RLE
Acid value, mg KOH/g	Initial	0.17 ± 0.01 ^a,b^	0.20 ± 0.0 ^d^	0.21 ± 0.01 ^e,f^
	3 months	0.17 ± 0.01 ^a,b^	0.20 ± 0.0 ^d^	0.21 ± 0.01 ^e,f^
	6 months	0.18 ± 0.0 ^b^	0.21 ± 0.01 ^e,f^	0.21 ± 0.01 ^e,f^
	9 months	0.19 ± 0.01 ^c,d^	0.21 ± 0.01 ^e,f^	0.22 ± 0.01 ^f,g^
	12 months	0.21 ± 0.01 ^e,f^	0.22 ± 0.01 ^f,g^	0.23 ± 0.0 ^g^
Peroxide value, mmol O_2_/kg	Initial	2.82 ± 0.04 ^c^	1.31 ± 0.06 ^a^	1.62 ± 0.05 ^b^
	3 months	3.05 ± 0.05 ^d^	1.53 ± 0.05 ^a,b^	1.95 ± 0.04 ^b^
	6 months	3.81 ± 0.02 ^e^	1.92 ± 0.07 ^b^	2.51 ± 0.01 ^c^
	9 months	4.64 ± 0.05 ^f^	2.56 ± 0.05 ^c^	2.93 ± 0.07 ^c^
	12 months	5.12 ± 0.06 ^g^	3.24 ± 0.08 ^d^	3.71 ± 0.05 ^e^
Conjugated diene content, µmol/g	Initial	8.60 ± 0.19 ^b^	7.20 ± 0.11 ^a^	7.25 ± 0.12 ^a^
	3 months	9.84 ± 0.17 ^d^	8.32 ± 0.17 ^b^	8.76 ± 0.11 ^b,c^
	6 months	11.32 ± 0.14 ^f^	8.98 ± 0.14 ^c^	9.51 ± 0.16 ^d,c^
	9 months	12.47 ± 0.18 ^g^	9.81 ± 0.18 ^d^	10.39 ± 0.18 ^e^
	12 months	13.66 ± 0.17 ^h^	10.95 ± 0.14 ^f^	11.31 ± 0.12 ^f^
Conjugated triene content, µmol/g	Initial	3.99 ± 0.04 ^b,c^	3.34 ± 0.04 ^a^	3.37 ± 0.03 ^a^
	3 months	4.64 ± 0.05 ^d^	3.92 ± 0.07 ^b^	4.13 ± 0.06 ^b,c^
	6 months	5.34 ± 0.03 ^f^	4.24 ± 0.05 ^c^	4.49 ± 0.07 ^c,d^
	9 months	5.88 ± 0.04 ^g^	4.63 ± 0.02 ^d^	4.90 ± 0.08 ^e^
	12 months	6.35 ± 0.05 ^h^	5.09 ± 0.08 ^e,f^	5.33 ± 0.09 ^f^
*p*-anisidine value, c.u.	Initial	0.848 ± 0.006 ^a,b^	0.841 ± 0.009 ^a,b^	0.844 ± 0.007 ^a,b^
	3 months	0.883 ± 0.009 ^c^	0.858 ± 0.006 ^b^	0.862 ± 0.006 ^b^
	6 months	0.928 ± 0.007 ^e^	0.898 ± 0.010 ^c,d^	0.902 ± 0.009 ^d^
	9 months	0.956 ± 0.008 ^f^	0.921 ± 0.009 ^d,e^	0.947 ± 0.005 ^f^
	12 months	1.087 ± 0.005 ^h^	0.986 ± 0.008 ^g^	0.991 ± 0.004 ^g^
β-Carotene, mg/100 g DW	Initial	nd	8.45 ± 0.95 ^b^	21.56 ± 0.04 ^e^
	3 months	nd	7.33 ± 0.62 ^b^	18.44 ± 0.78 ^d,e^
	6 months	nd	7.01 ± 0.78 ^a,b^	17.87 ± 0.52 ^d^
	9 months	nd	6.78 ± 0.67 ^a,b^	16.91 ± 0.68 ^c,d^
	12 months	nd	6.51 ± 0.50 ^a^	16.26 ± 0.82 ^c,d^
Lycopene, mg/100 g DW	Initial	nd	9.40 ± 0.06 ^b^	24.01 ± 0.95 ^f^
	3 months	nd	7.85 ± 0.32 ^b^	20.04 ± 0.93 ^d,e^
	6 months	nd	7.41 ± 0.64 ^a,b^	18.93 ± 0.56 ^d^
	9 months	nd	6.91 ± 0.71 ^a,b^	17.58 ± 0.89 ^c,d^
	12 months	nd	6.41 ± 0.89 ^a^	16.43 ± 0.78 ^c^
Zeaxanthin, mg/100 g DW	Initial	nd	9.55 ± 0.53 ^b^	24.36 ± 0.35 ^d^
	3 months	nd	8.28 ± 0.84 ^a,b^	20.83 ± 0.17 ^c^
	6 months	nd	7.89 ± 0.55 ^a,b^	20.04 ± 0.64 ^c^
	9 months	nd	7.54 ± 0.92 ^a,b^	19.62 ± 0.78 ^c^
	12 months	nd	7.35 ± 0.64 ^a^	18.38 ± 0.33 ^c^
β-Cryptoxanthin, mg/100 g DW	Initial	nd	8.87 ± 0.70 ^b^	22.68 ± 0.57 ^e^
	3 months	nd	7.43 ± 0.84 ^a,b^	19.01 ± 0.19 ^d^
	6 months	nd	7.16 ± 0.43 ^a,b^	17.58 ± 0.65 ^d^
	9 months	nd	6.71 ± 0.56 ^a,b^	16.11 ± 0.59 ^c^
	12 months	nd	6.26 ± 0.75 ^a^	14.96 ± 0.51 ^c^
Lutein, mg/100 g DW	Initial	nd	7.76 ± 0.05 ^e^	nd
	3 months	nd	6.52 ± 0.28 ^d^	nd
	6 months	nd	6.14 ± 0.41 ^c,d^	nd
	9 months	nd	5.58 ± 0.31 ^a,b^	nd
	12 months	nd	5.29 ± 0.23 ^a^	nd

nd = not detected; SLE = sea buckthorn lipophilic extract; RLE = rose hip lipophilic extract. Results are presented as mean value ± standard deviation. Different letters (^a–h^) designate statistically different results (*p* ≤ 0.05).

**Table 5:** Carotenoids used as standards in RP-HPLC analysis and their retention times.

Compound	Retention Time (min)	Max Absorption (nm)
Mutatoxanthin	7.572	400, 425, 448
Lutein	8.130	421, 445, 473
Zeaxanthin	10.406	426, 450, 476
β-Cryptoxanthin	37.622	428, 451, 476
*cis*-β-Carotene	70.144	424, 446, 472
all-*trans*-β-Carotene	75.181	421, 452, 478
γ-Carotene	83.695	434, 461, 488
Lycopene	98.317	448, 471, 503

## Data Availability

Not applicable.

## Supplementary Materials

The following supporting information can be downloaded at: https://www.mdpi.com/article/10.3390/molecules28083596/s1, Figure S1: UV-Vis spectra of liposoluble extracts from berries and sunflower oil depending on the extraction temperature: (A) sea buckthorn; (B) rose hip; Figure S2: FTIR spectra of liposoluble extracts from sea buckthorn (A) and rose hip (B) depending on the extraction temperature; Figure S3: Generalized fuzzy mathematical model PV = f(t, AV) for sunflower oil, sea buckthorn and rose hip extracts: (A) weights of fuzzy sets; (B) coefficients of the model; (C) the PV parameter, experimental values and from the fuzzy model.
