# Supplementary material for: Stabilization of Sunflower Oil with Biologically Active Compounds from Berries
Source: Molecules. 2023 Apr 20;28(8):3596. doi: 10.3390/molecules28083596 (PMC10143843; doi:10.3390/molecules28083596)
Supplement: Supplementary file 1 [file molecules-28-03596-s001.zip › molecules-2266076-supplementary.pdf]

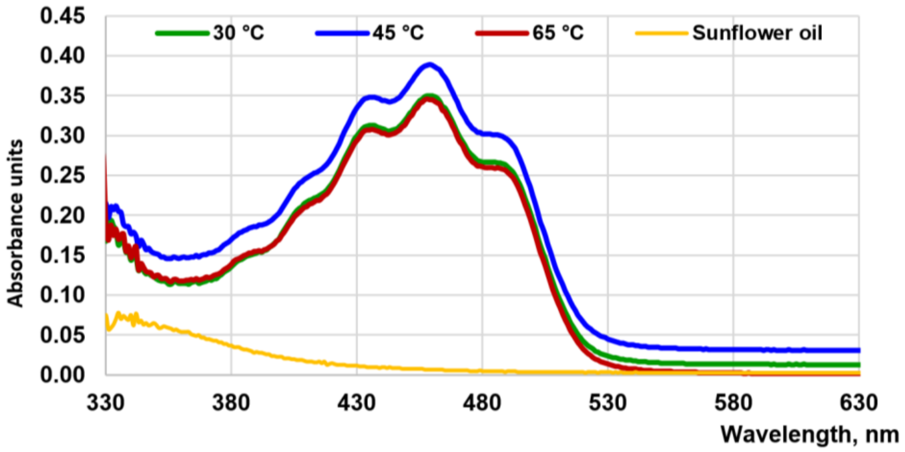

(A)

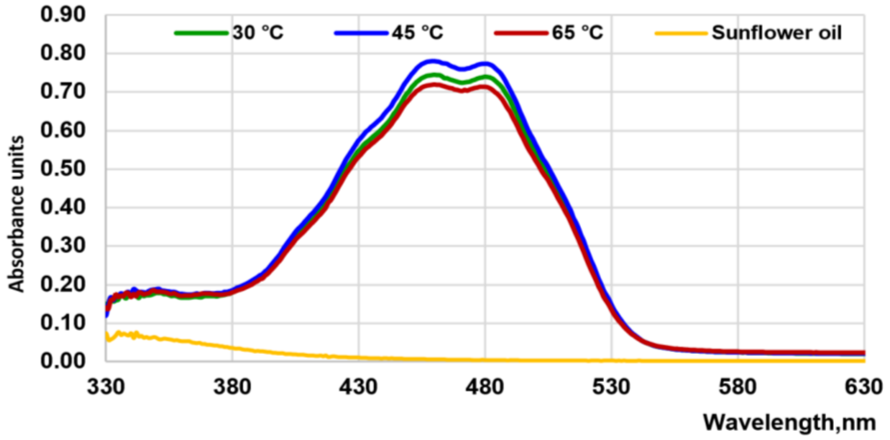

(B)

Figure S1. UV-Vis spectra of liposoluble extracts from berries and sunflower oil depending on the extraction temperature: (A) sea buckthorn; (B) rose hip.

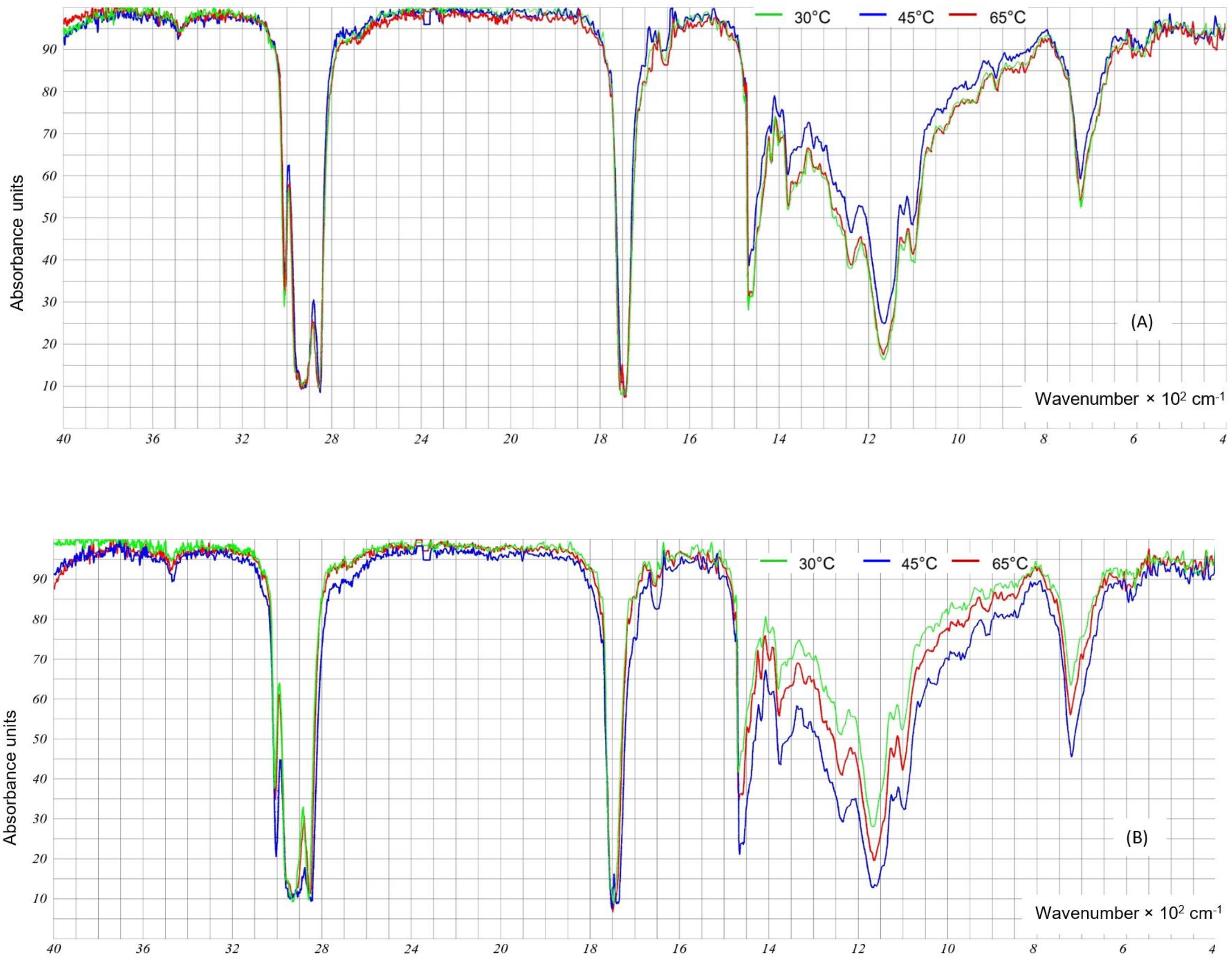

Figure S2. FTIR spectra of liposoluble extracts from sea buckthorn (A) and rose hip (B) depending on the extraction temperature.

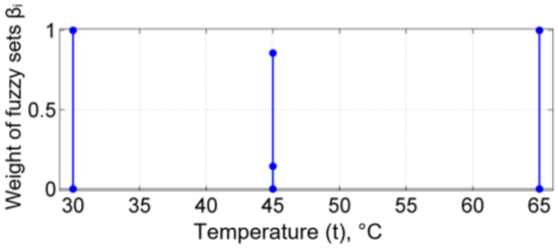

(A)

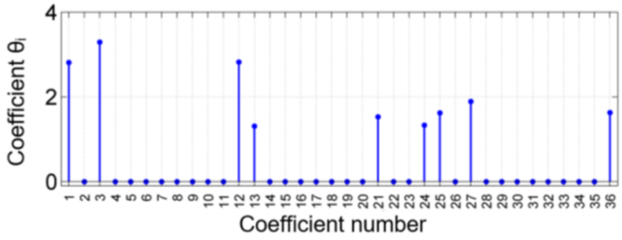

(B)

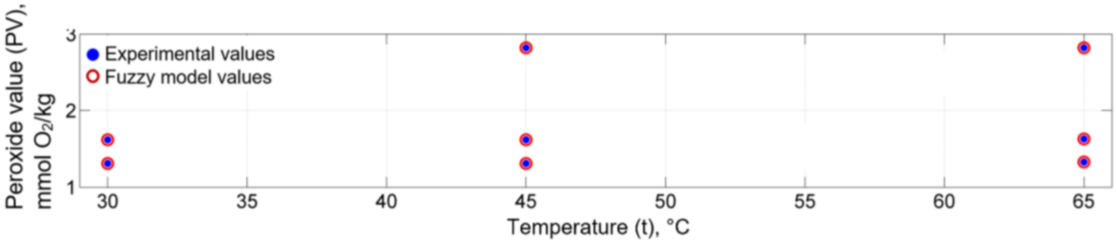

(C)

Figure S3. Generalized fuzzy mathematical model PV=f(t, AV) for sunflower oil, sea buckthorn and rose hip extracts: (A) weights of fuzzy sets; (B) coefficients of the model; (C) the PV parameter, experimental values and from the fuzzy model.
